# Detectability of fiducials’ positions for real‐time target tracking system equipping with a standard linac for multiple fiducial markers

**DOI:** 10.1002/acm2.13050

**Published:** 2020-10-15

**Authors:** Shunsuke Ono, Yoshihiro Ueda, Shingo Ohira, Masaru Isono, Iori Sumida, Shoki Inui, Masahiro Morimoto, Reiko Ashida, Masayoshi Miyazaki, Kazuhiko Ogawa, Teruki Teshima

**Affiliations:** ^1^ Department of Radiation Oncology Osaka International Cancer Institute Osaka Japan; ^2^ Department of Radiation Oncology Graduate School of Medicine Osaka University Osaka Japan; ^3^ Department of Cancer survey and gastrointestinal oncology Osaka International Cancer Institute Osaka Japan

**Keywords:** fiducial marker, real‐time target tracking, respiratory management, triggered image

## Abstract

**Purpose:**

To investigate the detectability of fiducial markers’ positions for real‐time target tracking system equipping with a standard linac. The hypothesis is that the detectability depends on the type of fiducial marker and the gantry angle of acquired triggered images.

**Methods:**

Three types of ball fiducials and four slim fiducials with lengths of 3 and 5 mm were prepared for this study. Triggered images with three similar fiducials were acquired at every 10° during the conformal arc irradiation to detect the target position. Although only one type of arrangement was prepared for the ball fiducials, a three‐type arrangement was prepared for the slim fiducials, such as parallel, orthogonal, and oblique with 45° to the gantry‐couch direction. To measure the detectability of the real‐time target tracking system for each fiducial and arrangement, detected marker positions were compared with expected marker positions at every angle of acquired triggered images.

**Results:**

For the ball**‐**type fiducial, the maximum difference between the detected marker positions and expected marker positions was 0.3 mm in all directions. For the slim fiducial arranged parallel and oblique with 45°, the maximum difference was 0.4 mm in all directions. When each slim fiducial was arranged orthogonal to the gantry‐couch direction, the maximum difference was 1.5 mm for the length of 3 mm, and 3.2 mm for the length of 5 mm.

**Conclusions:**

The detectability of fiducial markers’ positions for the real‐time target tracking system equipping with a standard linac depends on the form and insertion angles of the fiducials.

## 1 INTRODUCTION

To improve clinical results for pancreatic and prostate cancer, hypofractionation, stereotactic body radiation therapy, and microboosting to high‐risk areas are often performed.^1^ Hence, there is a growing significance for the motion management of tumors during irradiation.^2^, ^3^, ^4^, ^5^ In the chest, abdominal, and pelvis regions that have respiratory‐induced motion and physiological position errors, the bony anatomy matching results in clinically significant errors in the target localization and verification. To deliver radiation without geometric misses in these regions, implanted fiducials are useful because they help to predict the tumor’s position during irradiation.^6^, ^7^

To aid respiratory management, several systems for real‐time tumor tracking, such as Calypso (Varian Medical Systems, Palo Alto, CA) and Micropos (Micropos Medical AB, Goteborg, Sweden), are now available in clinics.^8^, ^9^, ^10^ More so, real‐time tumor‐tracking systems with oblique kV images for CyberKnife, O‐arm, and a standard linac were developed and applied in clinics.^11^, ^12^, ^13^, ^14^ In these systems, both kV‐sources and detectors were fixed on the floor and ceiling. Therefore, the angles of acquired kV images were constant during the treatments.

Recently, a novel real‐time target tracking system, namely the Auto Beam Hold (ABH), was developed with the on‐board imaging (OBI) of TrueBeam linear accelerator (Varian Medical Systems). This application allows the automatic detection of the positions of the implanted fiducials in the patient’s body with two‐dimensional kV images acquired by OBI during beam on. These acquired images are also referred to as triggered images in VMAT. The detected positions in the ABH systems are compared with the planned positions to determine in real‐time whether the beam stops or continues. The beam stops if the difference of marker position is greater than tolerance value, which is determined in the ABH setting. There have been several reports about the clinical effectiveness of ABH.^15^, ^16^

ABH has a limitation in that the visibility of slim fiducials among triggered images varies due to a change in the kV source and detector by rotating the gantry. There have been few discussions about the detectability of ABH in various types of fiducials. The geometric miss occurs due to a wrong detection of targets’ positions, and this decreases dose coverage for tumors.^17^ Moreover, a wrong detection of tumor positions in ABH may significantly increase treatment time as the ABH system does not allow to deliver irradiation without the images, which indicate that markers’ positions were correct.^14^, ^15^, ^16^ The aim of this study is to investigate the detectability of fiducial markers’ positions for real‐time target tracking systems, which are equipping with a standard linac. The study’s hypothesis is that the detectability depends on the type of fiducial markers, orientation of marker placement, and the gantry angle of acquired triggered images.

## 2 MATERIALS AND METHODS

### 2.A Fiducials and display settings

**Fig. 1:**
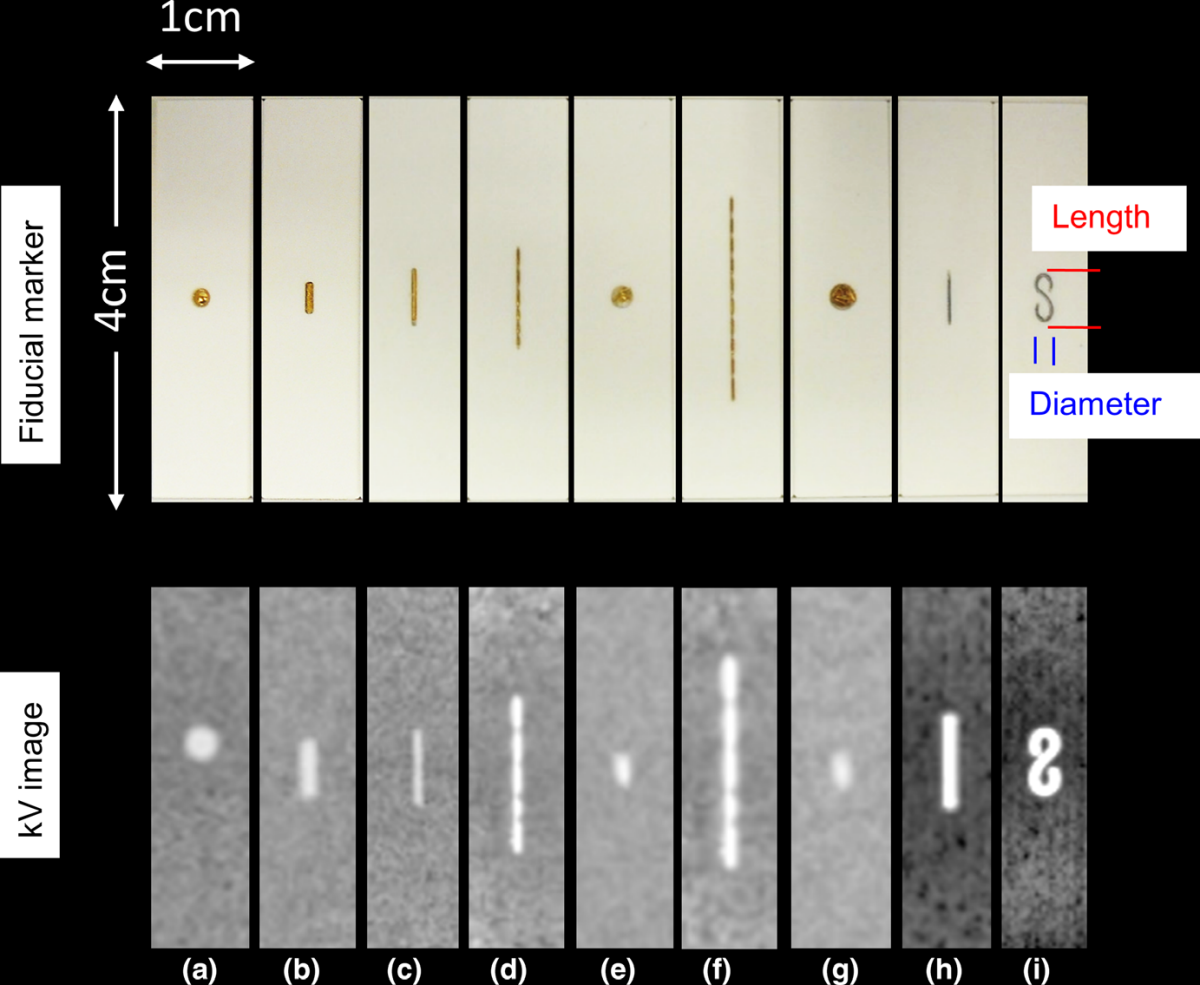
Nine types of fiducials embed in water‐equivalent phantom. The name, diameter as the interval between **blue lines** in mm, and length as the interval between **red lines** in mm for each fiducial are summarized in Table 1. (a) iGold (Medikit, Tokyo, Japan), 2, (b) Acculoc (Civco Medical Solutions, Iowa, USA), 1, 3. (c) VISICOIL (IBA, Bartlett, TN), 0.5, 5. (d) GoldAnchor of length in 10 mm, straight type (Naslund Medical AB, Huddinge, Sweden), 0.3, 10. (e) GoldAnchor of length in 10 mm, folded type (Naslund Medical AB), 2, 2. (f) GoldAnchor length in 20 mm, straight type (Naslund Medical AB), 0.3, 20. (g) GoldAnchor length in 20 mm, folded type (Naslund Medical AB), 2, 2. (h) LumiCoil straight type, (Boston Scientific, Boston, MA), 0.5, 5. (i) LumiCoil form 8 type, (Boston Scientific), 2, 5.

**Fig. 2:**
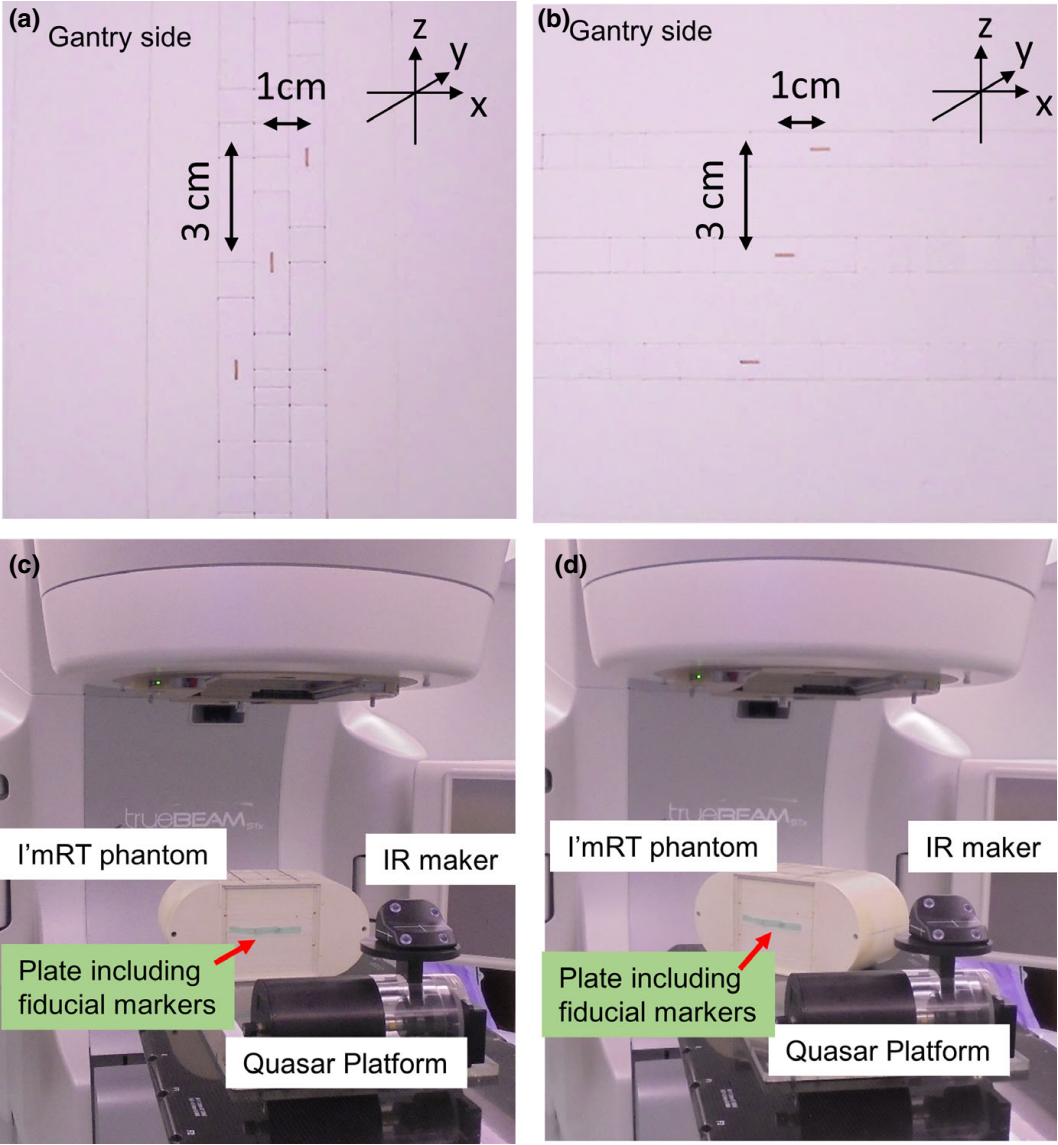
(a) Setting for fiducials. All fiducials turn to the gantry side. The distance of centers for fiducials was 3.16 cm ($\sqrt{3^{2}+1^{2}}$). (b) Experiment view. The I'mRT phantom and QUASAR platform were prepared on the treatment couch. The isocenter and the center of I'mRT phantom coincided. Infrared reflective marker was set on the QUASAR platform to use the respiratory management system of TrueBeam STx.

Nine types of fiducials were embedded on the surface of a cuboidal water‐equivalent phantom of size of 1.0 × 1.0 × 4.0 cm^3^ (Taisei Medical, Osaka, Japan) (Fig. 1). The center of the fiducials and cuboids coincided. In each fiducial, three similar cuboids were prepared. Table 1 summarizes the name, form, measured diameter, and measured length of each fiducial. As presented in Figs. 2(a) and 2(b), the plates with sizes of 16 × 16 × 1.0 cm^3^, including three similar fiducials, were created by displaying several sizes of cuboids with and without fiducials, and they were inserted in the I’mRT phantom (IBA Dosimetry, Bartlett, TN). The surface of the plate including fiducials coincided with the center of the I’mRT phantom.

**Table 1:** The form, measured, and set diameters and length in ABH for each fiducial.

Fiducial	Form	Measurement [mm]	Marker setting in ABH [mm]		
			Plate_A/A_45_	Plate_B	Plate_B
		Dia./Leng.	Dia./Leng.	Dia./Leng.	Dia./Leng.
iGold	Ball	2.0/2.0	2.0/2.0		
Acculoc	Slim	1.0/3.0	1.0/3.0	1.0/3.0	
VISICOIL	Slim	0.5/5.0	1.3/5.0	1.3/5.0	1.0/3.0
GA 10 mm	Slim	0.35/10			
GA 10 mm	Folded	2.0/2.0	2.0/2.0		
GA 20 mm	Slim	0.35/20			
GA 20 mm	Folded	2.0/2.0	2.0/2.0		
LC	Slim	0.45/5.0	1.3/5.0	1.3/5.0	1.0/3.0
LC	Slim (Form 8)	2.0/5.0	2.5/5.0	2.5/5.0	1.0/3.0

Abbreviations: Dia., Diameter; Leng., Length; ABH, auto beam holding; GA, GoldAnchor; LC, LumiCoil.

To simulate insertion angles of fiducials in a body, three settings were prepared. In the first setting (Plate_A), shown as Fig. 2(a), each slim fiducial turned to the gantry side. Here, the visibility of the fiducials does not change among triggered images acquired at several angles. In the slim fiducials, such as Figs. 1(b), 1(c), 1(h), and 1(i), the visibility of the triggered images depended on the gantry angles when the fiducials turned right or left. Thus, in the fiducials in Figs. 1(b), 1(c), 1(h), and 1(i), another setting (Plate_B) was prepared as shown in Fig.ure 2 (c). In Plate_B, each fiducial turn right or left. Plate_B represents fiducials which were inserted diagonally to the body’s axis. Finally, to simulate that when fiducials were inserted at 45° for a body axis, Plate_A_45_ was prepared as shown in Fig. 2(d). In Plate_A_45_, I'mRT phantom having the plate for Plate_A was set with 45° to the treatment couch. In Plate_A_45_, only VISICOIL (IBA, Bartlett, TN) was used.

### 2.B Beam settings

Conformal arc radiotherapy was used to acquire triggered images in Eclipse Ver 15.6 (Varian). During irradiation, the gantry rotated counterclockwise from 180° to extended 180°. The field size was 10 × 10 cm^2^, the irradiated total MU 900, and the dose rate 600 MU/min. The planned fiducial positions were determined manually. In each setting [Figs. 2(a) and 2(b)], the position of the center of each fiducial in (x mm, y mm, z mm) was (−1.0, 0, −3.0), (0, 0, 0), and (1.0, 0, 3.0). The coordinate of (0, 0, 0) represents isocenter.

The I’mRT phantom with the plate including fiducials, and the QUASAR platform (Modus Medical Devices, Ontario, Canada) with infrared reflective marker were placed on the treatment couch as shown in Fig. 2(b). The QUASAR platform was used to produce respiratory motion and acquire the gate signal for the ABH system. Therefore, the I'mRT phantom was not placed on the platform.

#### 2.B.1 ABH Settings for acquiring triggered images

During the experiments, the QUASAR platform moved sinusoidally to use the respiratory management system of TrueBeam STx (Varian Medical systems). In the amplitude gating, the gated region was set to cover the whole range of the trajectory to prevent the wave form from stopping the irradiation during the experiments. The treatment console was set to acquire the triggered images at every 10°. Therefore, they were acquired with 10° step from 90° of OBI kV source angle. The image acquisition parameters, such as tube voltage, tube current, and time, were 102–116 kVp, 100–172 mA, and 0.05–0.058 s, respectively.

In the ABH system, marker settings (diameter and length) and error tolerance should be determined before irradiation. Error tolerance value indicates the distance of detected marker position from planned marker position, which determine whether beams continue or stop. A straight type of GoldAnchors (Naslund Medical AB, Huddinge, Sweden) with lengths of 10 and 20 mm were excluded in this study as there was no setting for marker lengths >5 mm in the ABH system. In this study, to acquire all triggered images continuously during the arc even in the detection of setup errors, the error tolerance was deliberately made large, for example, making the diameter 3.0 cm. These parameters could also be changed during irradiation.

#### 2.B.2 Form of fiducials on triggered image

In Plate_A, a one‐marker setting was selected in the ABH system, and it is presented in Table 1 for each fiducial. In Plate_B, a two‐marker setting was selected in the ABH system for slim fiducials of length 5 mm. The marker length in the triggered images changed from diameter to length. For example, in VISICOIL, the marker length in the triggered images changed from 0.5 to 5 mm during the arc. When the marker length in the triggered images was <2 mm, a marker setting of 5 mm length in the ABH was inadequate. Hence, the ABH system could not detect the marker’s position. When the marker's length in the triggered images was approximately 5 mm, the marker setting in ABH was 3 mm in length, and the ABH system could not also detect the marker’s position. Therefore, two types of marker settings were prepared for Plate_B with VISICOIL and LumiCoil (Boston Scientific, Boston, MA) of length 5 mm. In Acculoc (Civco Medical Solutions, Iowa, USA) with a length of 3 mm, the marker length in the triggered images changed from 1 to 3 mm. Hence, a one‐type marker setting was adequate for Acculoc. In Plate_A_45_, changes of the observed length of fiducials in the triggered images were smaller than those of Plate_B. The marker length in the triggered images changed from 3.5 to 5 mm during one arc. The shortest length, 3.5 mm with the formula of 5/√2, was observed in the images acquired at angles of 90° and 270°. For Plate_A_45_, the same marker setting in Plate_A, such as a diameter of 1.3 mm and a length of 5.0 mm, was used.

### 2.C Evaluation of ABH system’s detectability

**Fig. 3:**
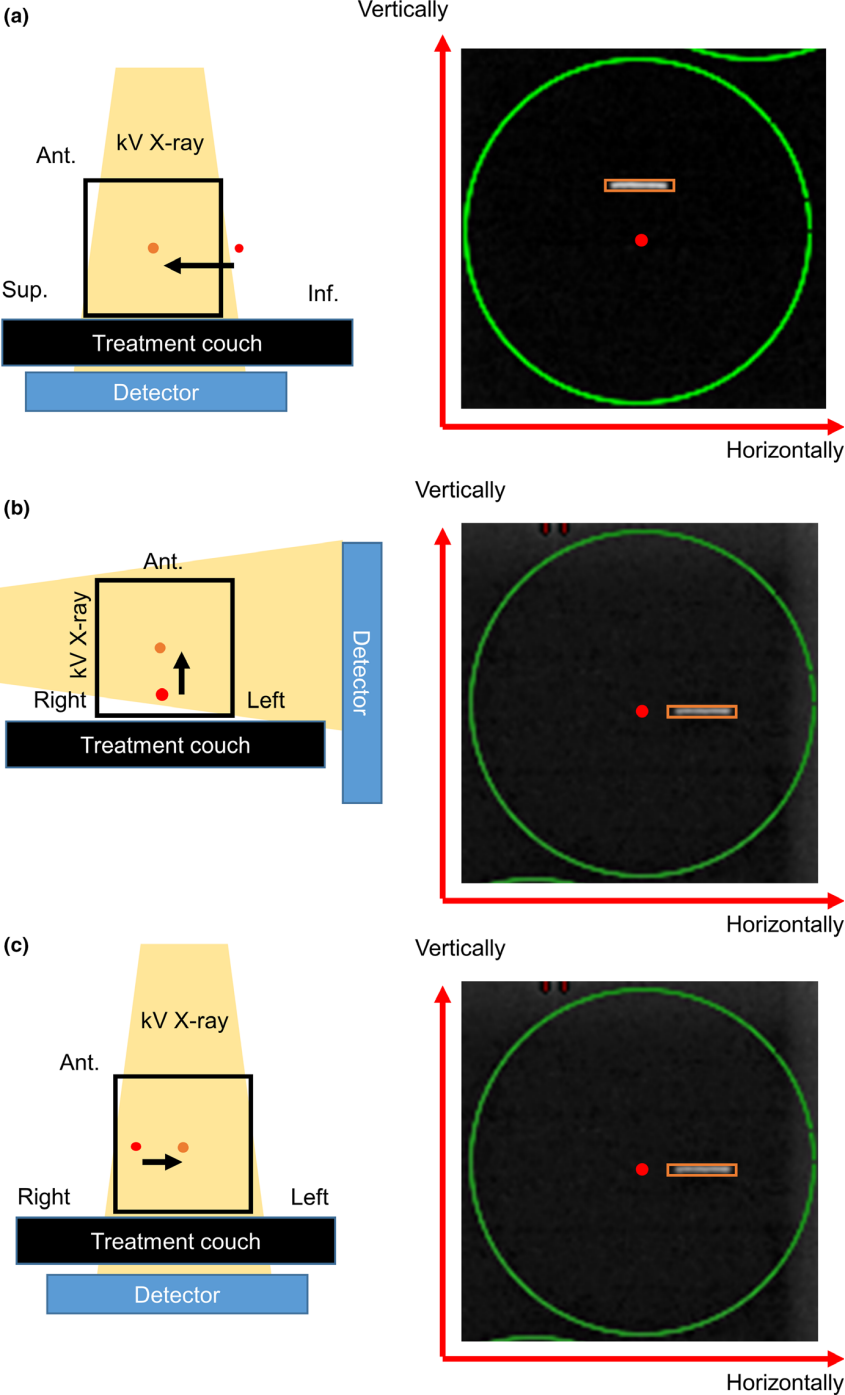
Shift of fiducial and triggered image. The **red** point represents isocenter, and the **orange** point the shift of fiducial. The **yellow** shade represents the kV x‐ray direction to the detector. In the triggered image, the **green circle** represents the tolerance of fiducial position error, the **red** point represents isocenter, and the **orange** square represents fiducial position. When fiducials move in the superior–inferior direction, the fiducials on the triggered image at the direction move vertically (a), when they move anterior‐posterior and right‐left, those on the triggered image move horizontally (b, c).

Before acquiring triggered images, the treatment couch was moved in anterior, left, or superior directions to simulate the setup error. Shifts of 5, 3, and 1 mm were selected in each direction. When the treatment couch moved in superior direction, the error was observed vertically in any triggered image [Fig. 3(a)]. The amount of error observed in the triggered images did not depend on the gantry angle and coincided with the shift value of the couch. When the treatment couch moved in the anterior or left directions, the setup error was observed in a horizontal direction in the triggered images [Figs. 3(b), 3(c)]. The amount of error observed in the triggered images depended on the gantry angle. For example, when the treatment couch moved 5 mm in the anterior direction, an error of 5 mm was observed horizontally in the triggered images acquired at 90° and 270°. However, no error was observed horizontally in the triggered images acquired at 0° and 180°. The expected setup error observed in the triggered images was calculated using the following trigonometric ratios for each angle.

When the setup error (d mm) occurred in the anterior direction, the following formula was used:

(1) $$\text{Expected value}=d\times \cos\left(90^{\circ }‐\theta \right)$$

When the setup error (d mm) occurred in the left direction, the following formula was used:

(2) $$\text{Expected value}=d\times \sin\left(90^{\circ }‐\theta \right)$$

where θ is the kV source angle of the triggered images.When the setup error (d mm) occurred in the superior direction, following formula was used:

(3) Expected value=d

The detected error of the fiducial position in each triggered image was recorded in a combined log file and extracted. This log file had detailed treatment information, such as coordinates of markers on the triggered images and the differences in fiducial positions. To measure the detectability of the ABH system for each fiducial, the detected values were compared with the expected values, which were calculated using the aforementioned formulas at the angles of the acquired triggered images.

The paired student's *t*‐test was used to examine the statistical significance differences in the detectability between following five settings.

1. GoldAnchor vs VISICOIL for Plate_A,
2. Plate_A vs Plate_B for Acculoc,
3. Plate_A vs Plate_B for VISICOIL,
4. Plate_A vs Plate_A_45_ for VISICOIL,
5. Appropriate setting vs inappropriate setting for ABH.

A *P* < 0.05 was considered to indicate statistical significance. All analyses were conducted with SPSS version 16 (SPSS Inc; Chicago, IL, USA).

## 3 RESULTS

**Fig. 4:**
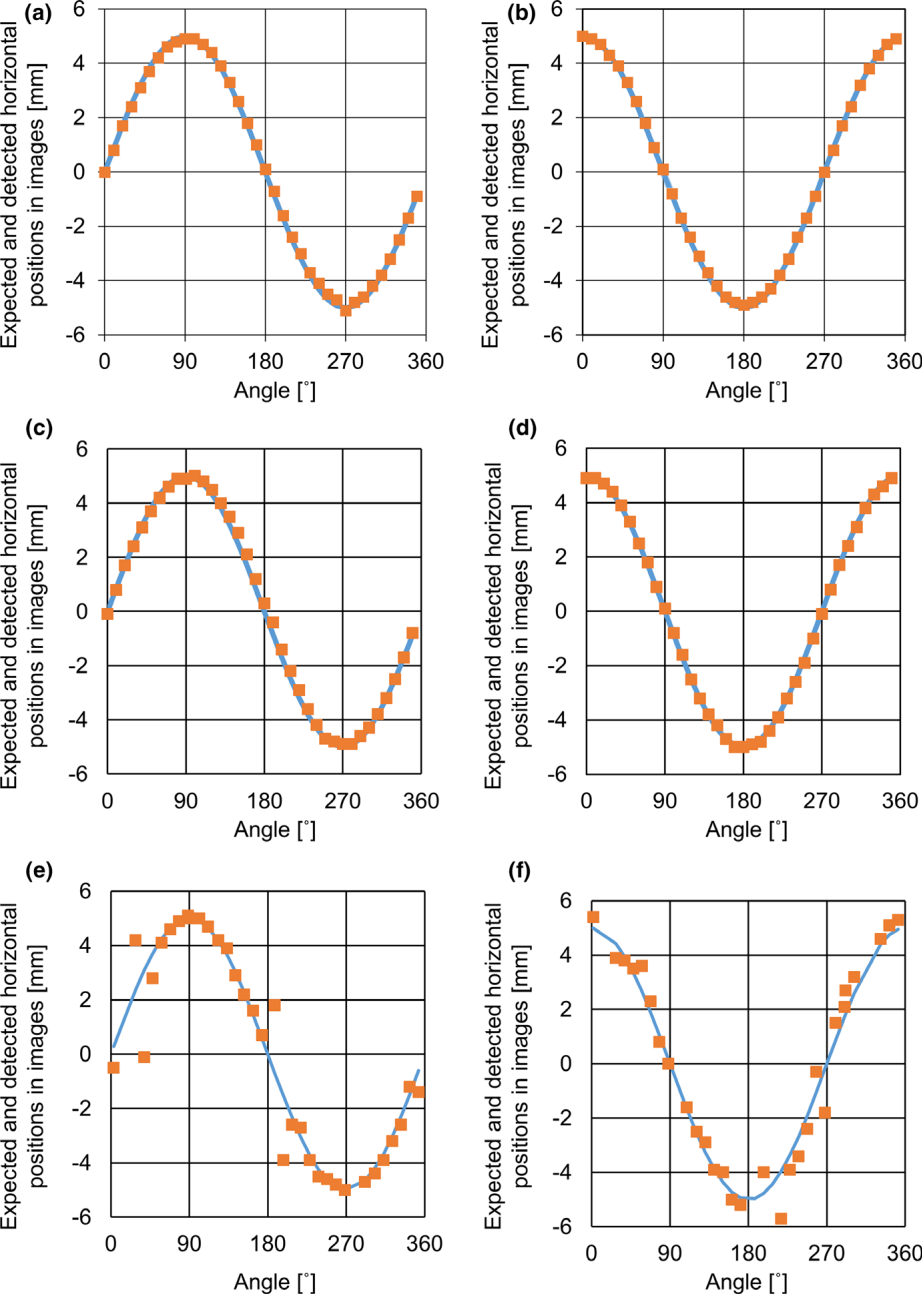
Comparisons between expected and detected horizontal positions for GoldAnchor, when setup error occurs in anterior direction (a) and left direction (d). The **blue** lines represent the expected horizontal positions, and the **orange** dots represent detected horizontal positions in the triggered images. Comparisons between expected and detected horizontal positions for VISICOIL in Plate_A, when setup error occurs in anterior direction (b) and left direction (e). Comparisons between expected and detected horizontal positions for VISICOIL in Plate_B, when setup error occurs in anterior direction (c) and left direction (f).

In the plate for Plate_A and Plate_B, Figure 4 shows the expected and detected positions in ABH when the fiducials had 5 mm of setup error in anterior and left directions. In Plate_A with GoldAnchor and VISICOIL, the expected and detected values were mutually consistent at all angles. Table 2 shows the absolute mean errors with each fiducial in the plate for Plate_A. In Plate_B with VISICOIL, the expected and detected values were mutually inconsistent at some angles.

**Table 2:** The mean value, standard deviation, and maximum values of differences in mm between the expected and detected positions in the triggered images when setup errors occurred for Plate_A in anterior, left, and superior directions.

Dir.	SE	Makers						
		iGold	Acculoc	VISICOIL	GA	GA	LC	LC
	Shift				10 mm	20 mm	Slim	Form 8
Ant.	5 mm	0.1 ± 0.1 (0.3)	0.3 ± 0.2 (0.4)	0.1 ± 0.2 (0.1)	0.0 ± 0.1 (0.1)	0.0 ± 0.1 (0.1)	0.1 ± 0.1 (0.3)	0.1 ± 0.1 (0.3)
	3 mm	0.1 ± 0.1 (0.2)	0.0 ± 0.1 (0.1)	0.1 ± 0.1 (0.1)	0.0 ± 0.1 (0.2)	0.0 ± 0.0 (0.1)	0.2 ± 0.2 (0.4)	0.1 ± 0.1 (0.4)
	1 mm	0.0 ± 0.0 (0.1)	0.0 ± 0.1 (0.2)	0.0 ± 0.1 (0.2)	0.0 ± 0.1 (0.1)	0.1 ± 0.1 (0.2)	0.1 ± 0.1 (0.3)	0.1 ± 0.1 (0.3)
Lt.	5 mm	0.1 ± 0.2 (0.3)	0.0 ± 0.2 (0.3)	0.0 ± 0.1 (0.2)	0.0 ± 0.1 (0.3)	0.1 ± 0.1 (0.1)	0.2 ± 0.2 (0.4)	0.1 ± 0.2 (0.3)
	3 mm	0.0 ± 0.1 (0.2)	0.0 ± 0.1 (0.1)	0.0 ± 0.1 (0.1)	0.1 ± 0.1 (0.2)	0.1 ± 0.1 (0.2)	0.1 ± 0.1 (0.3)	0.1 ± 0.1 (0.2)
	1 mm	0.0 ± 0.0 (0.1)	0.1 ± 0.1 (0.2)	0.0 ± 0.1 (0.1)	0.0 ± 0.0 (0.1)	0.1 ± 0.1 (0.2)	0.1 ± 0.1 (0.3)	0.1 ± 0.1 (0.3)
Sup.	5 mm	0.1 ± 0.2 (0.3)	0.1 ± 0.1 (0.2)	0.1 ± 0.1 (0.3)	0.2 ± 0.1 (0.3)	0.0 ± 0.1 (0.2)	0.1 ± 0.1 (0.3)	0.1 ± 0.1 (0.4)
	3 mm	0.1 ± 0.1 (0.2)	0.1 ± 0.1 (0.2)	0.1 ± 0.1 (0.3)	0.1 ± 0.1 (0.2)	0.1 ± 0.1 (0.2)	0.1 ± 0.1 (0.2)	0.0 ± 0.1 (0.2)
	1 mm	0.1 ± 0.1 (0.1)	0.1 ± 0.1 (0.2)	0.0 ± 0.1 (0.1)	0.1 ± 0.1 (0.1)	0.0 ± 0.1 (0.1)	0.1 ± 0.1 (0.2)	0.0 ± 0.1 (0.1)

Abbreviations: SE, setup errors; GA, GoldAnchor; LC, LumiCoil; Dir., Directions; Ant., anterior; Lt., left; Sup., superior.

Table 3 details the absolute mean errors with each fiducial in the plate for Plate_B. The maximum error was >2.0 mm in the anterior and left directions for fiducials, except in Acculoc where it was <1.5 mm. In the error in superior direction, the maximum error was <1.0 mm in all fiducials.

**Table 3:** The mean value, standard deviation, and maximum value of differences in mm between the expected and detected positions in the triggered images when setup errors occurred for Plate_B and Plate_A_45_ in anterior, left, and superior directions.

Dir.	SE	Plate_B				Plate_ A_45_
		Markers				
		Acculoc	VISICOIL	LC	LC	VISICOIL
	Shift			Slim	Form 8	
Ant.	5 mm	0.3 ± 0.2 (0.4)	0.0 ± 0.5 (0.9)	0.9 ± 1.1 (2.2)	0.4 ± 0.8 (3.2)	0.0 ± 0.1 (0.2)
	3 mm	0.3 ± 0.4 (0.9)	0.7 ± 0.9 (2.1)	0.7 ± 0.9 (2.4)	0.7 ± 1.1 (3.2)	0.0 ± 0.1 (0.2)
	1 mm	0.1 ± 0.1 (0.3)	0.1 ± 1.0 (2.7)	0.3 ± 0.5 (1.5)	0.5 ± 0.9 (2.8)	0.0 ± 0.1 (0.3)
Lt.	5 mm	0.0 ± 0.2 (0.3)	0.4 ± 0.7 (1.7)	0.7 ± 0.9 (3.1)	0.3 ± 0.5 (2.9)	0.0 ± 0.1 (0.3)
	3 mm	0.4 ± 0.5 (1.3)	0.7 ± 0.9 (2.8)	0.6 ± 0.9 (3.0)	0.5 ± 0.8 (3.0)	0.0 ± 0.1 (0.2)
	1 mm	0.1 ± 0.1 (0.3)	0.4 ± 1.0 (2.7)	0.4 ± 0.5 (1.5)	0.3 ± 0.6 (1.5)	0.0 ± 0.1 (0.3)
Sup.	5 mm	0.1 ± 0.1 (0.2)	0.0 ± 0.1 (0.1)	0.1 ± 0.1 (0.1)	0.1 ± 0.2 (0.8)	0.1 ± 0.1 (0.2)
	3 mm	0.1 ± 0.1 (0.2)	0.1 ± 0.1 (0.2)	0.1 ± 0.1 (0.3)	0.1 ± 0.2 (0.7)	0.1 ± 0.1 (0.3)
	1 mm	0.1 ± 0.1 (0.2)	0.1 ± 0.1 (0.2)	0.0 ± 0.1 (0.2)	0.1 ± 0.1 (0.3)	0.2 ± 0.1 (0.4)

Abbreviations: SE, setup errors; LC, LumiCoil; Dir., Directions; Ant., anterior; Lt, left; Sup., superior.

Table 4 shows the *P* values for differences in fiducial form and direction. There was no significant difference between GoldAnchor and VISICOIL for Plate_A. For slim fiducial, such as Acculoc and VISICOIL, there was significant difference between Plate_A and Plate_B.

**Table 4:** *P*‐values for each difference in fiducial type.

	*P*‐value
GoldAnchor vs. VISICOIL for Plate_A	.133
Plate_A vs. Plate_B for Acculoc	<.001
Plate_A vs. Plate_B for VISICOIL	<.001
Plate_A vs. Plate_A_45_ for VISICOIL	.614
Appropriate setting vs. inappropriate setting	<.001

**Fig. 5:**
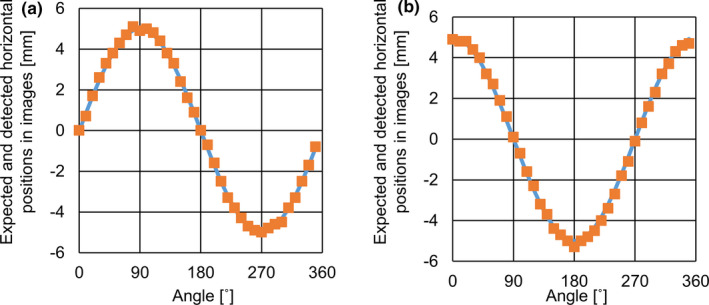
Comparisons between the expected and detected horizontal positions for VISICOIL in Plate_A_45_ when setup error occurs in anterior direction (a) and left direction (b). The **blue** lines represent the expected horizontal positions and the **orange** dots represent detected horizontal positions in the triggered images.

In Plate_A_45_, Fig. 5 shows the expected and detected positions in ABH when the fiducials had 5 mm of setup error in the anterior and left directions. The marker setting for the length and diameter in ABH was the same as that of the experiment with Plate_A. In Plate_ A_45_, the expected and detected values were mutually consistent at all angles. Table 3 shows the absolute mean errors with each fiducial in the plate for Plate_ A_45_. In each fiducial and direction, the absolute difference was <0.5 mm. There was no significant difference between Plate_A and Plate_A_45_ for VISICOIL.

**Fig. 6:**
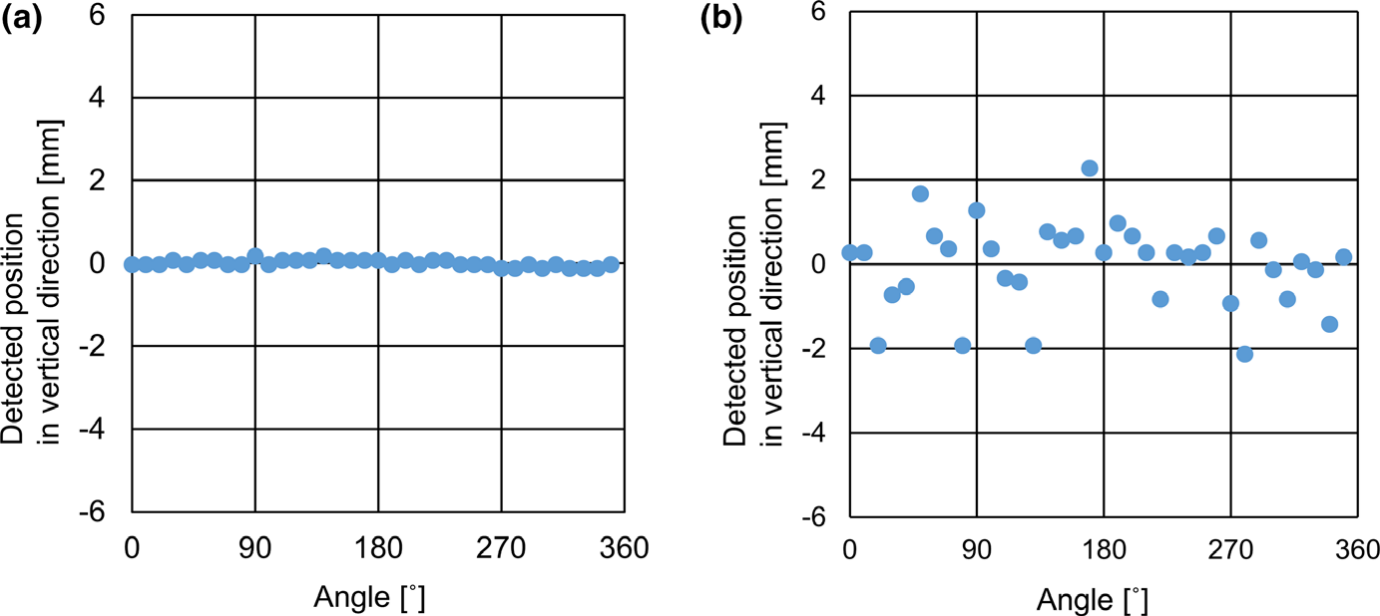
Detected vertical positions in the appropriate (a) and inappropriate settings (b) when VISICOIL was used in the plate for Plate_A. The appropriate setting was 5 mm in length in the ABH system. The inappropriate setting was 3 mm in length in the ABH system. There were no setup errors in any direction. **The blue** dots were of the vertical position in the triggered images detected in the ABH system.

Figure 6 shows the differences between the expected and detected positions in both appropriate and inappropriate ABH settings when VISICOIL, such as fiducial length of 5.0 mm, was used for Plate_A. In the appropriate settings of length 5 mm, the mean absolute error was 0.1 ± 0.0 mm, and the maximum absolute error was 0.2 mm. In the inappropriate settings, the mean absolute error was 0.8 ± 0.6 mm, and the maximum absolute error was 2.3 mm. There was significant difference between appropriate setting and inappropriate setting.

## 4 DISCUSSIONS

The detection of fiducials’ positions in a body using a linac equipped with an imaging system is novel without previous investigations. The visibility of some fiducials in the triggered images changed during irradiation because the acquired angles of the triggered images changed by rotating the gantry for VMAT. Some fiducials, such as iGold and folded GoldAnchor, did not change the length of triggered images when the fiducial’s form was ball or approximately ball. The results show that these fiducials have a high accuracy in any insertion angle. When users use the slim fiducials, they should pay attention to the changing length of fiducials in the triggered images. In this study, it was verified that changing the visibility of the fiducials affected the detection of the positions in the ABH system. Further, this study suggests optimal fiducials and insertion angles of fiducials in a body for the ABH system.

The insertion angle of fiducials in Plate_A was parallel to the body’s axis. As presented in Table 2, the maximum error is 0.4 mm for Plate_A in all fiducials. When the insertion angle of the fiducials was easily parallel to the body’s axis, it was found that the detectability of the fiducials’ position with the ABH system was highly accurate for each fiducial. In previous studies involving prostate cancers, the insertion angle of the fiducials implanted from the perineum was found to be easily parallel with the body’s axis,^6^, ^18^ and the angle of the fiducials implanted from the rectum was easily oblique with the body’s axis. Rosario et al.^15^ also used the ABH system for VMAT in prostate cancers, precisely the 1.2 mm × 5 mm gold marker (QLRAD, Zwolle, The Netherlands). The conditions used by these researches are likely similar to those of Plate_A and Plate_A_45_ using VISICOIL. In prostate cancer, the detectability of the ABH will be accurate for any fiducials.

Furthermore, it was believed that the insertion angle of fiducials was perpendicular to the body’s axis in a transcutaneous placement of fiducials in the liver, because short path for the insertion is better for patients. In patients placed with VISICOIL on liver at our institution, the insertion angles for fiducials were 90° ± 10° in 10%. The insertion angles are similar to those of Plate_B. In the settings, the changing lengths of the fiducials in the triggered images were more pronounced than in Plate_A and Plate_A_45_. Therefore, in Plate_B of the slim fiducials, there were angles at which the length of fiducials in the triggered images significantly differed from the setting length in the ABH system. At these angles, the detectability of ABH reduced swiftly. When the slim fiducials in Plate_B were used, users needed to change the settings of fiducials in ABH during the arc. However, in Plate_B, short slim fiducials, such as Acculoc, had smaller errors than the other slim fiducials, such as VISICOIL. This was due to the smaller changing lengths of the short slim fiducials in the triggered images compared to the slim fiducials. To prevent a detection of positions in errors due to the changing lengths in triggered images, we recommend using short slim fiducials for the ABH system.

The ABH system can simultaneously detect three fiducials’ positions. However, only one fiducial setting is determined in the system. In the clinics, the length of three fiducials in the triggered images was different in each case when slim fiducials were used, as in Fig. 1, in a report by Vinogradskiy et al.^16^ Hence, it is possible that some fiducials were detected normally while others were detected with inappropriate settings. In the latter case, users may remove the fiducials with lengths that do not match with the marker setting in ABH.

Slim fiducials, such as VISICOIL and LumiCoil, may bend in the body. Bended coils for VISICOIL have been observed in clinics. In this study, there was no bended fiducials, except LumiCoil with form 8. Therefore, it was not evaluated whether the ABH system moved normally to the bended fiducials. As detailed in Plate_B in Table 2, the difference between the expected and detected positions in the vertical direction was <0.5 mm in all fiducials except in LumiCoil with form 8 when the setup error occurred in superior direction. The reason is that slim coils were described as lines or points in the triggered images. The length of these lines and points was vertically <1 mm. However, LumiCoil with form 8 was described as “∞” or “▮” and the length of “∞” and “▮” was vertically 2 mm at maximum. Hence, LumiCoil with form 8 in Plate_B has larger errors than any fiducials in vertical directions. When fiducials bend in a body, a change in the thickness occurs in the triggered images. Hence, the detected errors become larger. Therefore, it is recommended that fiducials do not bend in a body.

Regarding limitations, this study examined the detectability of fiducial position using only phantom images rather than clinical images. Three fiducials did not overlap with one other during irradiation, and the inside of the phantom did not include inhomogeneous materials in this measurement. In clinics, fiducials inserted in a body may overlap with bones and other organs by the angle of acquiring the triggered images. Additionally, the image acquisition parameters vary depending on the body’s thickness, and the optimal parameters for each patient are not clear.^19^

## 5 CONCLUSIONS

The detectability of fiducial markers’ positions with the ABH system depends on the visibility of the fiducials in the triggered images. To ensure high detectability of the ABH system, the following are recommended. First, fiducial markers should be inserted in the body such that the angles that change the visibility of the fiducials are small in triggered images. Second, short slim or ball fiducial markers should be used.

## CONFLICT OF INTEREST

The authors declare that they have no conflict of interest.
